# Trends in use of the new MeSH term “overdiagnosis”: A bibliometric review

**DOI:** 10.1111/hir.70000

**Published:** 2025-08-09

**Authors:** Emma Grundtvig Gram, Barnett S. Kramer, Karsten Juhl Jørgensen, Steven Woloshin

**Affiliations:** ^1^ Center of General Practice, Department of Public Health University of Copenhagen Copenhagen Denmark; ^2^ Lisa Schwartz Foundation for Truth in Medicine Norwich Vermont USA; ^3^ Cochrane Denmark and Centre for Evidence‐Based Medicine Odense University of Southern Denmark Sønderborg Denmark Denmark; ^4^ The Dartmouth Institute for Health Policy and Clinical Practice Lebanon New Hampshire USA

**Keywords:** indexing, medical subject headings (MeSH), MEDLINE, overdiagnosis, review, bibliometrics, subject headings

## Abstract

**Objectives:**

Although the concept of overdiagnosis was first referenced in MEDLINE 100 years ago, consensus on a clear definition has been lacking. In 2021, the MeSH term “Overdiagnosis” was officially introduced, which defined the concept. A key goal of the new term is to improve the reliability of literature searches and enhance the conceptual understanding of overdiagnosis.

**Methods:**

We conducted a systematic bibliometric review of all citations indexed under the MeSH term for “Overdiagnosis” in MEDLINE. We compared the citations with citations identified through a text‐word search for overdiagnosis not indexed under the MeSH term. Searches were performed on 15 September 2024.

**Results:**

We found that a higher percentage of citations indexed under the new MeSH term used it according to the definition compared with the text‐word search (73.2% vs. 49.5%). The remainder used the term to describe misdiagnosis, false positives, and overtreatment. The citations indexed under the MeSH term were primarily descriptive in nature (68.7%), focusing on oncology (54.2%) and screening practices (31.2%).

**Discussion:**

Despite advancements, the field of overdiagnosis is still in its early stages, with potential for expansion into studies addressing prevention and mitigation strategies. The introduction of the MeSH term has facilitated some degree of conceptual alignment.

**Conclusion:**

Our review provides insights into the current state of the overdiagnosis literature, emphasising prevalent themes and areas for further research, and improvements in MeSH indexing accuracy. Residual conceptual ambiguity surrounding overdiagnosis terminology and indexing practices may explain discrepancies in MeSH categorisation and definition adherence.

**Key Points**

- The MeSH term “Overdiagnosis” in MEDLINE defines the concept, fostering consensus.
- Some citations misuse “overdiagnosis” for misdiagnosis, false positives, or overtreatment; MeSH indexing needs refinement for accuracy.
- The “Overdiagnosis” MeSH term aids organising literature and facilitates research on overdiagnosis.

## INTRODUCTION

The Medical Subject Headings (MeSH) term “Overdiagnosis” was officially introduced in 2021 to refine conceptual consensus and support systematic searching and indexing of research on overdiagnosis (Woloshin & Kramer, 2021). MeSH terms are controlled and defined by the National Library of Medicine and are used for indexing, cataloguing, and searching biomedical and health‐related information. Until 2011, every publication was manually indexed by people working at the National Library of Medicine. After 2019, the National Library of Medicine experimented with full computer‐automated indexing, incorporating human review of select citations including concepts of known ambiguities and random samples of citations (NLM, 2023). In 2024, machine‐learning models were implemented for indexation trained by citations published between 2007 and 2022, assigning MeSH terms based on statistical likelihood (NLM, 2024). In 2021, the average time from publication to MeSH assignment was 145 days, while today it is typically done within one business day (NLM, 2023). The Medical Text Indexer‐neXt Generation (MTIX) algorithm makes determinations based on many features and not just trigger words and thus can index citations based on abstract concepts not explicitly present in the text. Human indexers regularly perform quality assurance review of automatically indexed citations. Today, human curators review one‐third of articles indexed via automation, focusing their efforts on areas with high impact, such as clinical trials and systematic reviews (NLM, 2024).

The new MeSH definition of overdiagnosis refers to technically correct but clinically unhelpful diagnoses (Box 1). By this definition, overdiagnosis is distinct from misdiagnosis, false positives, and overtreatment.

Our aim is to review the use of the term and track the development over time in citations published under the MeSH term “Overdiagnosis” in MEDLINE. This review fills a gap identified in an earlier review from 2017, which found that definitions of overdiagnosis were diverse across medical literature and lacked consensus (Jenniskens et al., 2017). We aim to map the characteristics of the use of the concept of overdiagnosis post‐development of the formal dedicated MeSH term.

BOX 1 **MeSH term definition of “Overdiagnosis”.**

“*The labeling of a person with a disease or abnormal condition that would not have caused the person harm if left undiscovered, creating new diagnoses by medicalizing ordinary life experiences, or expanding existing diagnoses by lowering thresholds or widening criteria without evidence of improved outcomes. Individuals derive no clinical benefit from overdiagnosis, although they may experience physical, psychological, or financial harm*.” (1).

### Overdiagnosis historically

**FIGURE 1:**
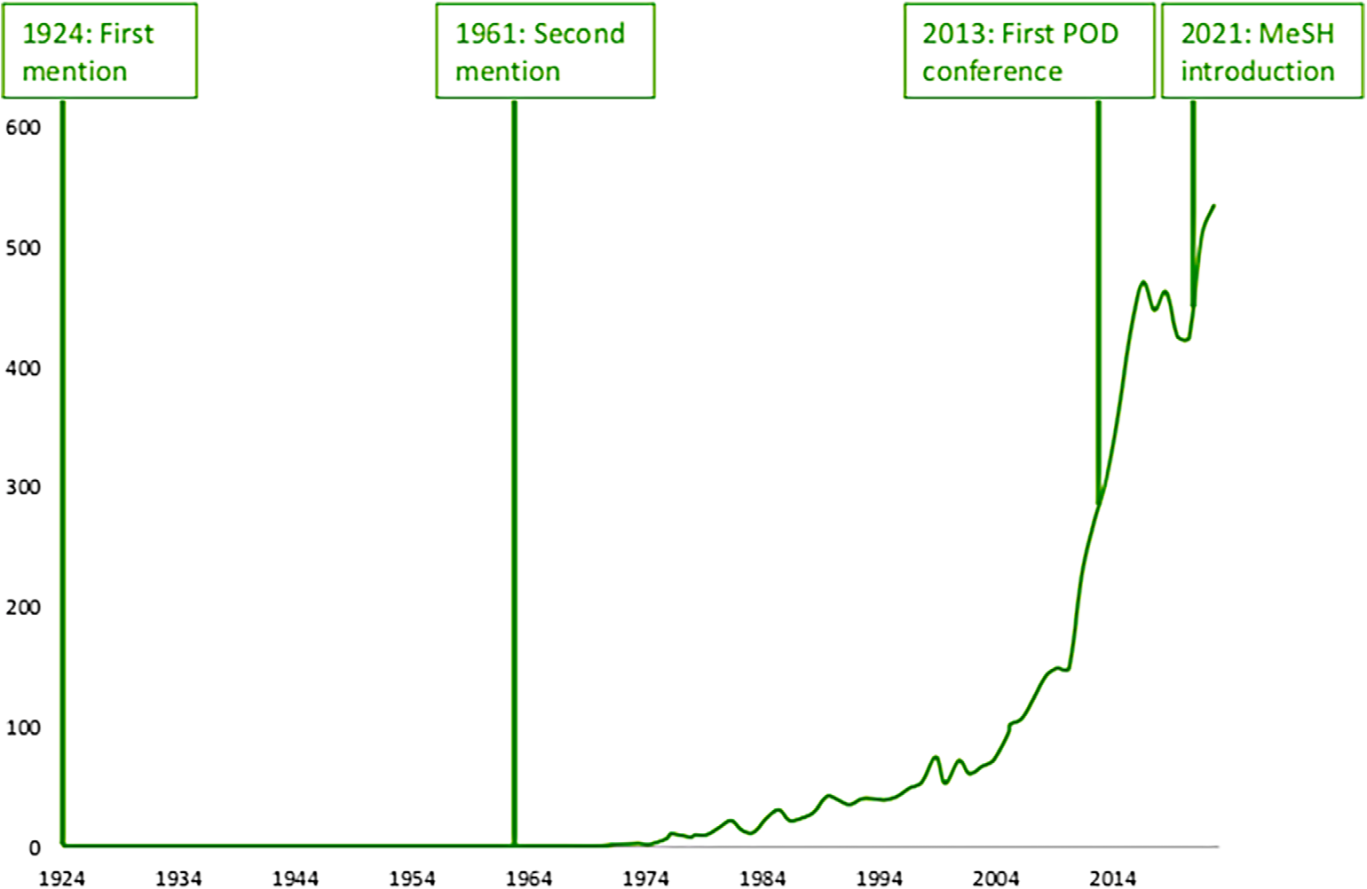
Number of citations mentioning overdiagnosis (count by year). Search query: (Overdiagnosis(MeSH Terms)) OR (Overdiagnos*(Text Word)). [Colour figure can be viewed at wileyonlinelibrary.com]

The first paper that mentioned overdiagnosis was published 100 years ago (Adamson, 1924). The paper described how increased focus on early detection of tuberculosis would diagnose cases of tuberculosis without the presence of tuberculosis bacteria (Adamson, 1924). The authors termed this overdiagnosis but would, in today's usage, be classified as misdiagnosis. Possibly, there have been earlier notions of overdiagnosis, but using other terms to describe the concept. The second mention was 37 years later in 1961, describing the potential under‐ and overdiagnosis in routine examination of photofluorograms for tuberculosis (Wijkstrom, 1961), also what we today would classify as misdiagnosis. From around 1980, an exponential growth in the number of citations mentioning overdiagnosis was observed (Figure 1). Reflecting—and perhaps stimulating—interest in overdiagnosis. In 2013, the first official international conference on overdiagnosis was held at the Dartmouth Institute in collaboration with the British Medical Journal (BMJ) and the Centre for Evidence Based Medicine Oxford: the Preventing Overdiagnosis Conference. Conferences have been held every one to two years in subsequent years.

For a long time, there has been variation in the use of the term overdiagnosis, including definition, confusions with other terms such as misdiagnosis and false positives, and adaptation of the term to apply to new disease areas. This has been evident at conferences and in formal and informal literature searches in attempts to track research interest in the topic (Jenniskens et al., 2017). Such informal searches also showed that searches were unreliable as some known relevant papers were not found, while some irrelevant ones were. Together with the growing interest and literature, this conceptual variation prompted the creation of a dedicated MeSH term for overdiagnosis. The formal MeSH term introduction in 2021 represents a step towards enhanced indexing accuracy, coherence, and comparability of research, and a shared conceptual understanding of overdiagnosis.

## METHODS

The protocol for this review is published on the Open Science Forum (Gram et al., 2023). We used the BIBLIO checklist for reporting this bibliometric review (Montazeri et al., 2023). We performed two separate searches: one based on the new MeSH term for Overdiagnosis and one based on a text‐word search in PubMed/MEDLINE. Text‐word searches include title, abstract, headings, publication type, and authors (PubMed User Guide, 2024). We included every publication labelled under the new MeSH term. From the text‐word search, given the large number of items identified, we used an online randomisation tool to select a 10% random sample of all citations published after June 2020 which contained the word “overdiagnosis” and were not indexed using the MeSH term. June 2020 was the date the first citation was indexed in MEDLINE under the new MeSH term and thus this month was chosen as the inception time for the text‐word search to make searches comparable in that regard. Our searches were performed on 30 March 2023 and updated on 15 September 2024.

We iteratively developed and refined codes to maximise reliability, and to capture the characteristics of the identified citations. We test coded citations from the text‐word search that were not included in this review. All authors coded the same three articles, compared codes, discussed disagreement, and revised code definitions. This process was repeated over four iterations, at which point few disagreements occurred. Next, 30% of all included citations were double coded by blinded and independent assessors. After agreement of codes, the remaining citations were coded independently by a single reviewer. Ambiguous citations and codes were noted and discussed within the author team. We aimed to estimate Kappa statistics for inter‐rater reliability using standard thresholds (Landis & Koch, 1977). However, there were only five disagreements for 235 codes (2.2%). Our agreement was thus high. In such cases, Kappa becomes unstable, so it was not calculated (Feinstein & Cicchetti, 1990). Disagreements on the double‐coded citations were resolved within the author team at regular meetings.

Data on the year of publication, first author, title, name of the journal, and language were automatically extracted from PubMed. Data on publication type, topic, aim, and disease area were assessed manually based on the abstract of each indexed publication using the final codes presented in Table 1.

**TABLE 1:** Data extraction from the two searches.

	MeSH search	Text‐word search^a^
	*N* = 227	*N* = 109
Disease area	*N* (%)	*N* (%)
Oncology	123 (54.2%)	51 (46.4%)
Non‐specific/multiple	29 (12.8%)	8 (7.4%)
Endocrinology	18 (7.9%)	5 (4.6%)
Infectious diseases	13 (5.7%)	14 (12.8%)
Cardiology	12 (5.4%)	8 (7.4%)
Muscolosceletal	10 (4.4%)	7 (6.2%)
Psychiatry	8 (3.5%)	9 (8.3%)
Neurology	7 (3.1%)	2 (1.8%)
Rheumatology	3 (1.3%)	1 (0.9%)
Immunology	3 (1.3%)	3 (2.8%)
Ophtalmology	1 (0.4%)	1 (0.9%)
Topic		
Screening	71 (31.2%)	40 (36.7%)
Medical overuse	46 (20.2%)	21 (19.3%)
Other^b^	35 (15.4%)	9 (8.3%)
Test accuracy	30 (13.2%)	25 (23%)
Disease definition	23 (10%)	11 (10%)
Time trends	23 (10%)	3 (2.7%)
Aim		
Descriptive (eg., general issues)	156 (68.7%)	88 (80.8%)
Estimating prevalence of overdiagnosis ODx	40 (17.7%)	7 (6.4%)
Interventional efforts to reduce overdiagnosis	25 (11%)	14 (12.8%)
Other^c^	6 (2.6%)	0 (0%)
About overdiagnosis		
Yes	166 (73.2%)	54 (49.5%)
No	61 (26.8%)	55 (51.5%)
Not about overdiagnosis	*N* = 61 (26.8%)	*N* = 55 (50.5%)
Misdiagnosis	37 (60.7%)	12 (22%)
False positive	13 (21.3%)	14 (25.5%)
Overtreatment	10 (16.4%)	4 (7%)
Non‐related concepts^d^	1 (1.6%)	25 (45.5%)

^a^ Citation included the word “overdiagnosis” in title or text; but the citation was not indexed under the MeSH header “overdiagnosis”.

^b^ Examples include “biological risk” of overdiagnosis and overdiagnosis of invasive cancer.

^c^ Examples include reducing misdiagnosis.

^d^ Examples include evaluation of non‐attendance in cancer screenings, lifestyle and risk of cancer, and test accuracy.

## RESULTS

Between 1924 and 2022, a total of 5524 citations have mentioned overdiagnosis anywhere in the text, including about 600 added in 2022 (Figure 1). Only about 3% of these were indexed under the new MeSH term.

From June 1, 2020 to September 15, 2024, we identified 227 citations that were indexed under the MeSH term for overdiagnosis. Our text‐word search identified 1085 citations that used the term overdiagnosis, from which a 10% random sample of 109 citations was drawn. Results of the citations identified through the two searches are presented in Table 1.

The citations indexed under the new MeSH term were dominated by descriptive studies (68.7%) and oncology dominated the disease categories (54.2%), while screening dominated the topic categories (31.2%). The screening category included all aspects of screening, for example, disease awareness, self‐examination, screening performed by a physician, and incidental findings.

Of citations identified through the text‐word search showed parallel trends regarding disease areas and topics but identified a higher proportion of studies on test accuracy (23.0%) when compared with the MeSH search (13.2%). The test accuracy category included studies on test bias, sensitivity, and specificity. The text‐word search featured a higher proportion of descriptive studies (80.8% vs. 68.7%) and a lower proportion of studies that estimated the prevalence of overdiagnosis (6.4% vs. 17.7%).

**FIGURE 2:**
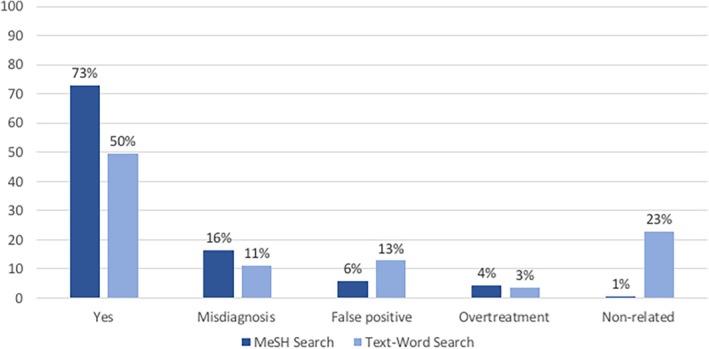
Use of “overdiagnosis” in citations: Percentage matching the MeSH definition (yes) vs. alternative meanings. [Colour figure can be viewed at wileyonlinelibrary.com]

73.2% of the citations indexed under the MeSH term used the term overdiagnosis in accordance with the National Library of Medicine definition, compared with 49.5% of citations identified in our text‐word search (Figure 2). Most citations (60.7%) indexed under the MeSH term that were not about overdiagnosis described misdiagnosis, 21.3% described false positives, and 16.4% overtreatment, and one citation was not about overdiagnosis at all (1.6%) (Table 1). Of the citations identified through the text‐word search that were not about overdiagnosis, 25.5% were about false positives, 22.0% were about misdiagnosis, 7.0% were about overtreatment, and 45.5% addressed topics that were completely unrelated to overdiagnosis (Table 1). These non‐related topics included identifying key genes linked to cancer, the impact of lifestyle on cancer risk, drug allergy, and factors associated with non‐attendance in screening.

Box 2 shows the top 10 journals in which studies indexed under the MeSH term for overdiagnosis were published. Annals of Internal Medicine was the most popular journal among studies on overdiagnosis, while the list also included the British Medical Journal (BMJ) and two additional BMJ journals: BMJ Evidence‐Based Medicine and BMJ Open. The JAMA Network was also represented on the list with their main journal and their dermatology journal.

BOX 2 **Top 10 journals citations for publication of MeSH‐indexed overdiagnosis citations.**

1. Annals of Internal Medicine
2. Clinical Orthopaedics and Related Research
3. The British Medical Journal (BMJ)
4. BMJ Evidence‐Based Medicine
5. European Urology
6. American Family Physician
7. JAMA Dermatology
8. Journal of the American Medical Association (JAMA)
9. Journal of Clinical Epidemiology
10. BMJ Open

## DISCUSSION

### Future for overdiagnosis research and the MeSH term

The historical trajectory of the use of the term overdiagnosis reveals an exponential increase in use since the 1980s, signifying a growing awareness and acknowledgment within academia. However, the absence of specific MeSH terminology is an impediment to a comprehensive and systematic assessment of any rapidly expanding body of medical literature. It was for this reason that the term “overdiagnosis” was proposed and accepted as a new MeSH term in 2021. The introduction of the new MeSH term offered the opportunity to conduct a “natural experiment” to explore trends and accuracy in its use. Our review revealed that the literature was predominantly descriptive and strongly dominated by studies of cancer and screening. It is to be expected that, as the concept of overdiagnosis is increasingly recognised in the clinical literature, the use of the MeSH term will further expand to other fields such as psychiatry, neurology, rheumatology, dermatology, and even dentistry (Nadanovsky et al., 2024).

The current focus on oncology in overdiagnosis research may be attributed to the widespread population screening for many cancers and the concerns about potential harms. The dominance of descriptive studies suggests that the field of overdiagnosis is still in its formative stage; while descriptive studies are foundational for understanding any problem, they represent the initial step before the problem can be addressed. The ultimate goal is to progress beyond description towards the development of interventions to prevent overdiagnosis or to mitigate its harms. While some efforts have already been made, our findings motivate attention to the need for more interventional research efforts to reduce overdiagnosis (O'Connor et al., 2023; Ropers et al., 2021; Miller, 2020; Welch et al., 1999).

An important effect of MeSH listing is not solely to increase awareness of emerging medical concepts and to facilitate systematic assessments of the literature, but also to increase precision in use of terminology. In fact, we found that there were fewer citations that were not about overdiagnosis, according to the National Library of Medicine definition, in the MeSH search compared with the text‐word search. In other words, the MeSH term was more specific. In the MeSH search, misdiagnosis was the most common conceptual confusion with overdiagnosis, which was also the case for the first and second papers to mention overdiagnosis in 1924 and 1961 (Adamson, 1924; Wijkstrom, 1961). The majority of citations, however, were about overdiagnosis according to the official definition, indicating that the introduction of the MeSH term contributes to conceptual consensus. The new MTIX algorithm used by the National Library of Medicine is able to recognise abstract concepts that are not explicitly present in the text and also distinct different usage of concepts (NLM, 2024). Theoretically, the algorithm could be trained to distinct different definitions or usage of overdiagnosis to use for indexation of the MeSH term. Nevertheless, 24% of citations indexed under the MeSH term were not about overdiagnosis, and therefore the indexation of this MeSH term could be further improved in terms of accuracy. This result also underlines the need for improved precision in the understanding and use of terminology within academia. As the field of overdiagnosis matures and expands, it would therefore be important to assess sensitivity and specificity in use of the term over time and in a broad spectrum of disease categories. Development of high specificity and sensitivity search filters, as has been done in other domains (e.g., systematic reviews), would be helpful (Lefebvre et al., 2013). The National Library of Medicine processes updates to MeSH terms annually (NLM, 2025).

### Implications for indexing practices and database searching

Our study has identified one important way to increase sensitivity of the new MeSH term: During the coding of studies, we found that few studies used the spelling “over‐diagnosis” (with a hyphen). Even though the entry terms for the MeSH term do include overdiagnosis in a hyphenated form, overdiagnosis unhyphenated seems to be the most popular spelling by far. A study on palliative care noted that studies hyphenating relevant words or concepts are more likely to be missed in systematic searches (Damarell & Tieman, 2016). This identifies challenges in information retrieval of studies on conditions that some investigators or word processors hyphenate while others do not. For now, researchers should include “over‐diagnosis” in systematic searches to avoid missing relevant literature. Subheadings spelled with and without a hyphen should be considered in information system design and management of health information services. This finding has implications for the approach of health information specialists to ensure the accuracy and effectiveness of electronic retrieval, and systematic reviews should use both the MeSH term and a text‐word search to capture all relevant literature. Combining text‐words and MeSH terms has also proven more successful than individually, along with the introduction of other MeSH terms such as the one for Evidence‐Based Medicine in 1997 (Harrison, 1997). Using both text‐word and MeSH terms has generally proven to increase precision of systematic searches (DeMars & Perruso, 2022; Golder et al., 2006; Lu et al., 2009). The National Library of Medicine does not retrospectively index MEDLINE citations with new MeSH terms (NLM, 2025). This means that searching for the overdiagnosis MeSH term would typically be limited to citations indexed after the introduction of the MeSH term in 2021 (NLM, 2025). Therefore, text‐words should still complement systematic searches to capture incorrectly and non indexed citations.

There might also be limitations to the computer‐automated algorithm used for indexing or the methodology employed in database indexing that needs refinement. Statistical likelihood of a MeSH term based on title, abstract, publication year, and journal name might not be correct, as we have also identified in our search. The National Library of Medicine aims to, in the future, expand algorithms to include the full text, which might improve indexation. Users can identify whether a citation was indexed fully by human, automatically, or curated (algorithmically with human review) through the XML record for the given citations under IndexingMethod (NLM, 2023).

The registration of the MeSH term “overdiagnosis” serves as a practical tool for organising and categorising medical reports, especially with the exponential growth in literature (Hunter & Cohen, 2006). Other researchers have emphasised the use of MeSH terms for systematic searches due to its internationally recognized standard, frequent updates, and widespread use in universities and medical libraries (Flor et al., 2001). Furthermore, it helps to validate overdiagnosis as a research term and can lead the way to further refinement. It reflects a recognition within academia of the relevance and significance of the problem. Further, the use of the MeSH term provides a formal definition and may improve further conceptual consensus (Nelson & Schulman, 2010).

Although sample size might be relatively small, this review points to trends in overdiagnosis literature and identifies gaps and ways forward.

### Implications for bibliometric analyses

Other scholars have used MeSH terms to study research trends (Lu & Bianchi, 2020; Lyu et al., 2015; Wang et al., 2017). Yang & Lee have also proposed a new method for identifying and visualising trends in the use of existing and emerging MeSH terms across a range of research fields (Yang & Lee, 2018).

A bibliometric review examined the MeSH term frequency and correlation with other MeSH terms and found that MeSH term indexing is very inclusive and might falsely index studies that are not appropriate for the specific MeSH term (DeShazo et al., 2009). The results of our study confirm and update that finding: a number of studies indexed under the MeSH term for overdiagnosis were actually about overtreatment, misdiagnosis, or false‐positive results; that is, inaccurate diagnoses rather than true overdiagnosis (which refer to technically correct but clinically unhelpful diagnoses). Another study found that the algorithm employed by the National Library of Medicine has an imbalance between the number of citations indexed with the MeSH (true positives) and the number of citations not indexed (false negatives) (Yepes et al., 2013). This suggests that the model currently used by the National Library of Medicine has a tendency to under‐index relevant studies ahead of the risk of false positives. However, the National Library of Medicine has updated their algorithm since these findings, and the new MTIX correctly applies more than 50% more terms than previous models (NLM, 2024). We found that the MeSH‐based search had a lower percentage of studies that were not about overdiagnosis (false positive) than the text‐word search. However, we also found that many citations about overdiagnosis were not indexed under the MeSH term, suggesting (false negative). A bibliometric review on knowledge synthesis methods in PubMed also found inconsistencies in MeSH indexing, including wrong MeSH indexing (false positives), and that relevant MeSH terms were often not applied to studies (Perrier et al., 2016). The study, however, suggests that such inconsistency can be due to studies that are published before the introduction of relevant MeSH terms, which is supported by the fact that the National Library of Medicine does not typically perform retrospective indexing of citations with new MeSH terms. Time will tell whether this is equally true in the emerging field of disease overdiagnosis.

### Implications for research

Researchers who would like to make sure that their work is indexed under the MeSH term for overdiagnosis can, first of all, make sure that the word is mentioned in the title or in the abstract since current indexation does not use full‐text information (NLM, 2024). Researchers who would like to explore if the MeSH term “overdiagnosis” is relevant to their manuscript can consult the MeSH Browser or visit MeSH on demand (Medical Subheadings, 2023). Researchers could take into account that the unhyphenated form of spelling overdiagnosis seems to be the most used.

Users are encouraged by the National Library of Medicine to provide feedback and report potential indexing errors through their Customer Service Link (NLM, 2023). Researchers whose papers about overdiagnosis are not indexed correctly could use this service.

Since there is no dedicated journal for overdiagnosis, the top 10 lists of journals might help researchers direct their research towards journals that focus on overdiagnosis or publish research on overdiagnosis. For example, The BMJ has launched a “Too Much Medicine” series, and JAMA a “Less is More” series. The top 10 list of journals also shows that overdiagnosis research is published in high‐ranking journals such as the BMJ, Annals of Internal Medicine, and JAMA.

### Implications for health

Overdiagnosis continues to be an important threat to health. While overdiagnosis has direct consequences for people who are overdiagnosed going through unnecessary treatment, psychosocial consequences, labeling effects, worry, and financial costs, overdiagnosis also has an impact on health care providers, society, and the planet (Harris et al. 2013). Health systems are under pressure, lacking resources, including time, personnel, and finances, to tackle increasing demand for health care (Johansson et al., 2023). Further, health care services are a well‐known cause of greenhouse gas emissions, leading to climate change, soil degradation, and ecosystem disruption. Emissions from healthcare account for about 5% of global emissions (Lenzen et al., 2020). At least 10–30% of these could be avoided if we stopped doing things that had a negative benefit‐harm ratio (Braithwaite et al., 2020). Overall, continuous efforts to prevent or reduce overdiagnosis are much needed. Current research endeavours to prevent or reduce overdiagnosis or low‐value care include active surveillance (Lončar et al., 2021; LORIS, 2024), clinician‐targeted feedback interventions (O'Connor et al., 2023), shared‐decision making efforts, de‐intensifying screening (Miller, 2020; Ropers et al., 2021), developing an understanding of cancer biology (Brawley & Ramalingam, 2023), developing guidelines to avoid over‐testing (Theriault & Grad, 2024), and teaching overdiagnosis to medical students (Colbert et al., 2024).

## CONCLUSION

Our review provides insights into the current state of overdiagnosis literature. It highlights the predominant themes and identifies research gaps and challenges in MeSH indexation accuracy. We observed that the overdiagnosis literature was dominated by descriptive studies with a focus on oncology and screening. Further, we noted that only a small proportion of studies reported on solutions or interventions to reduce or prevent overdiagnosis. We identified conceptual confusion about overdiagnosis terminology and indexing practices that might explain why some studies on overdiagnosis are not indexed under the MeSH term and why some studies that do not use the term according to the official definition are indexed. These findings have the potential to improve indexation practices and systematic searches on overdiagnosis as the field matures. This overview of overdiagnosis literature also points to ways forward for reducing and preventing overdiagnosis. Current efforts include active surveillance, shared‐decision making interventions, teaching medical staff, and developing guidelines to prevent overuse.

## FUNDING INFORMATION

This study did not receive any funding.

## CONFLICT OF INTEREST STATEMENT

The authors have no interests to declare.
